# Supplementary material for: Ultrasmall and tunable TeraHertz surface plasmon cavities at the ultimate plasmonic limit
Source: Nat Commun. 2023 Nov 23;14:7645. doi: 10.1038/s41467-023-43394-w (PMC10667513; doi:10.1038/s41467-023-43394-w)
Supplement: Supplementary file 1 — Supplementary Information [file 41467_2023_43394_MOESM1_ESM.pdf]

# Supplementary information

## Ultrasmall and tunable TeraHertz surface plasmon cavities at the ultimate plasmonic limit

Ian Aupiais[1], Romain Grasset[1], Tingwen Guo[1], Dmitri Daineka[2], Javier Briatico[3], Sarah Houver[4], Luca Perfetti[1], Jean-Paul Hugonin[5], Jean-Jacques Greffet[5], Yannis Laplace[1]

[1] LSI, CEA/DRF/IRAMIS, CNRS, Ecole Polytechnique, Institut Polytechnique de Paris, Palaiseau, France

[2] LPICM, CNRS, Ecole Polytechnique, Institut Polytechnique de Paris, Palaiseau, France

[3] Unité Mixte de Physique, CNRS, Thales, Université Paris Saclay, 91767 Palaiseau, France

[4] Université Paris Cité, CNRS, Matériaux et Phénomènes Quantiques, F-75013, Paris, France

[5] Université Paris-Saclay, Institut d'Optique Graduate School, CNRS, Laboratoire Charles Fabry, 91127 Palaiseau, France

# Contents

**1 Experimental determination of optical and geometrical parameters** **2**
1.1 Bulk parameters of the THz plasma of InSb . . . . . 2
1.2 Insulator permittivity . . . . . 3
1.3 Geometrical parameters of the samples . . . . . 3
1.4 Determination of the nonlocal parameter $v_F$ . . . . . 4

**2 Patch m/i/p THz cavity** **5**
2.1 Patch m/i/p cavity experimental results . . . . . 5
2.2 THz light confinement from stripe m/i/m to patch m/i/p resonators . . . . . 6

**3 Analysis of THz plasmonic modes in periodic lattices of m/i/p cavities** **7**
3.1 Dispersion diagram . . . . . 7
3.2 Interpretation based on Fabry-Perot resonances of m/i/p and vacuum/i/p waveguides . . . . . 8
3.3 Intuitive picture . . . . . 9

**References** **11**

# 1 Experimental determination of optical and geometrical parameters

## 1.1 Bulk parameters of the THz plasma of InSb

The frequency dependent permittivity $\epsilon_p(\nu)$ of bulk InSb is modelled with the Drude formula:

$$\epsilon_p(\nu) = \epsilon_\infty \left(1 - \frac{\nu_p^2}{\nu(\nu + i\Gamma)}\right)$$

where $\nu$, $\nu_p$, $\Gamma$ and $\epsilon_\infty$ are the frequency, plasma frequency, carrier scattering rate and background permittivity of the material, respectively. By performing time-domain THz spectroscopy on a bulk InSb sample (referenced to a Gold coated portion of the sample, see methods), we determine the absolute values of both the power and the phase of the reflected THz signal and compare them with theoretical predictions from the Drude model. The best fits used for the determination of $\nu_p$, $\Gamma$ and $\epsilon_\infty$ as a function of temperature are shown in Supplementary Fig. 1.

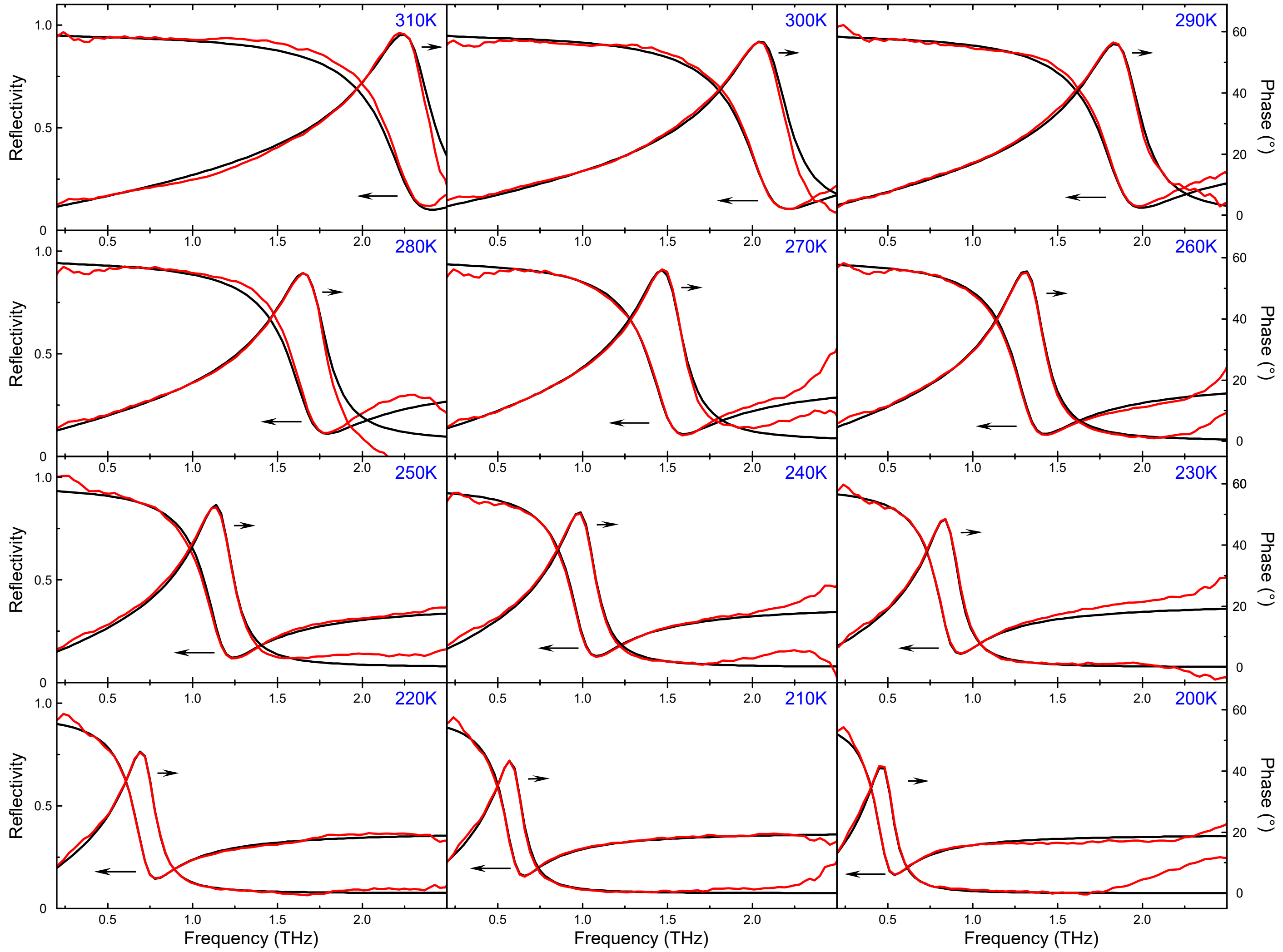


**Supplementary Figure 1:** Panels displaying the experimental power reflectivities and phases of the THz reflectivity of bulk InSb (red solid lines) and the fits obtained from the Drude model (black solid lines). The corresponding temperatures are labeled in blue.

The temperature dependence of $\nu_p$ and $\Gamma$ is already reported in Fig. 1 of the main text. As for $\epsilon_\infty$, we determine $\epsilon_\infty = 17.5 \pm 1.0$, a value that is independent of temperature within the uncertainty. Let's note that this background permittivity takes into account the ionic contribution of the IR-active optical phonon mode located at 5.4 THz in this material. We find our value of $\epsilon_\infty$ to be in full agreement with that previously reported [1]. Within our frequency window, frequency dispersion of $\epsilon_\infty$ given by the ionic contribution of the high-frequency optical phonon can safely be neglected as it amounts to less than 3%, i.e. is below our experimental resolution. We conclude that the permittivity of InSb shows excellent agreement with the Drude formula over the whole temperature and frequency ranges investigated.

## 1.2 Insulator permittivity

To determine the permittivity of the deposited $Si_3N_4$ insulator, we manufactured a sample consisting of a periodic lattice of striped metal/insulator/metal (**m/i/m**) cavities. Starting from a bulk substrate, the sample consists in 200 nm of Aluminum (doped with 1% Copper), the insulating $Si_3N_4$ and a Ti(15 nm)/Au(200 nm) stripe grating. Grating parameters and insulator thickness were measured to be $s = 52.0\ \mu m \pm 0.2\ \mu m$, $a = 18.0\ \mu m \pm 0.2\ \mu m$ and $d = 3.09\ \mu m \pm 0.02\ \mu m$. As seen in Supplementary Fig. 2, this sample exhibits a single resonance corresponding to the fundamental mode of the **m/i/m** cavity. We performed numerical EM-simulations of this sample with the permittivity of $Si_3N_4$ left as a free parameter to be determined and by taking permittivities of metallic Gold [2] and Aluminum [3] in the THz frequency range. We find the resonance frequency of the cavity to be sensitively dependent on the insulator permittivity, allowing us to determine $Re(\epsilon_{Si_3N_4}) = 6.1 \pm 0.2$, a value which is consistent with previous reports [4, 5]. Importantly, the permittivity is independent of temperature as can be seen temperature-independent position of the resonance in the spectra of Supplementary Fig. 2a. Additionally, the dispersion of the permittivity as well as the imaginary part can be safely neglected in our frequency range [5].

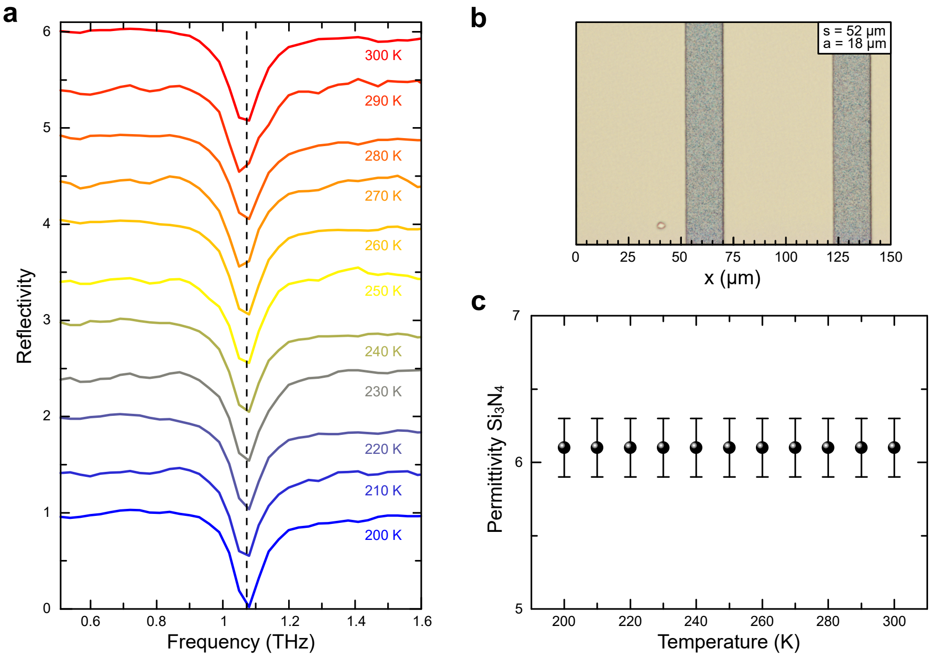


**Supplementary Figure 2:** **a**, Experimental reflectivity spectra of the stripe **m/i/m** cavity sample. The spectra are offset for clarity. **b**, Top view optical picture of the sample. **c**, Experimental determination of the temperature dependence of the permittivity of $Si_3N_4$.

## 1.3 Geometrical parameters of the samples

The geometrical parameters $s$ and $a$ of the samples were determined from optical and electron microscopy. The insulator thickness $d$ was determined from ellipsometry and profilometry. These parameters are reported in Supplementary Tab. 1.

| | Sample 1 | Sample 2 | Sample 3 | Sample 4 | Sample 5 | Sample 6 |
|---|---|---|---|---|---|---|
| Type | Stripe **m/i/p** | Stripe **m/i/p** | Stripe **m/i/p** | Stripe **m/i/p** | Patch **m/i/p** | Stripe **m/i/m** |
| $s$ | 41.0 $\mu m$ $\pm$0.2 $\mu m$ | 31.2 $\mu m$ $\pm$0.2 $\mu m$ | 20.7 $\mu m$ $\pm$0.2 $\mu m$ | 5.5 $\mu m$ $\pm$0.1 $\mu m$ | 5.0 $\mu m$ $\pm$0.1 $\mu m$ | 52.0 $\mu m$ $\pm$0.2 $\mu m$ |
| $a$ | 16.0 $\mu m$ $\pm$0.2 $\mu m$ | 11.8 $\mu m$ $\pm$0.2 $\mu m$ | 8.2 $\mu m$ $\pm$0.2 $\mu m$ | 1.5 $\mu m$ $\pm$0.1 $\mu m$ | 2.0 $\mu m$ $\pm$0.1 $\mu m$ | 18.0 $\mu m$ $\pm$0.2 $\mu m$ |
| $d$ | 2.95 $\mu m$ $\pm$0.01 $\mu m$ | 2.96 $\mu m$ $\pm$0.01 $\mu m$ | 2.96 $\mu m$ $\pm$0.01 $\mu m$ | 0.240 $\mu m$ $\pm$0.015 $\mu m$ | 0.240 $\mu m$ $\pm$0.015 $\mu m$ | 3.09 $\mu m$ $\pm$0.02 $\mu m$ |

**Supplementary Table 1:** Geometrical parameters of the samples

### 1.4 Determination of the nonlocal parameter $v_F$

Nonlocality is simulated in this study with the local analogue model (LAM) introduced in [6]. It consists in simulating the interface with a nonlocal medium by inserting an analogue material of very small thickness $h$ and local permittivity $\epsilon_{LAM}$ given by:

$$\epsilon_{LAM}(\omega) = \frac{hq_L\epsilon_p\epsilon_\infty}{\chi_f}$$

where $\epsilon_p(\omega) = \epsilon_\infty\left(1 - \frac{\omega_p^2}{\omega(\omega+i\Gamma^*)}\right) = \epsilon_\infty + \chi_f$ is the permittivity of the medium for the transverse components of the EM-fields, $\chi_f = -\frac{\epsilon_\infty\ \omega_p^2}{\omega(\omega+i\Gamma^*)}$ is the susceptibility, $q_L = \frac{\sqrt{\omega_p^2-\omega(\omega+i\Gamma^*)}}{\beta}$ $(Re(q_L) \geq 0)$ is the longitudinal plasmon wavevector and $\beta = \sqrt{\frac{3}{5}}\, v_F$. The carrier velocity $v_F$ is the only parameter describing the physics of nonlocality that enters in the computation.

To validate this method in the context of our study, we compare in Supplementary Fig. 3 the profile $|E_y|^2$ of the electric field normal to the interface for a TM-polarized plane wave incident at 80° on a planar $Si_3N_4$/InSb interface in the following two cases :

- for a rigorous nonlocal computation taking into account boundary conditions imposed on the continuity of the longitudinal and transverse components of the EM-fields as described in [7] and [8] (hard-wall boundary conditions in the present case),
- for a simulation of the LAM with our RCWA code.

We observe that the rigorous nonlocal computation and the LAM computation only differ in the vicinity of the interface. The small kink observed close to the interface (located $y = 0\ \mu m$) in the rigorous nonlocal computation is due to the finite penetration depth of the longitudinal component of the electric field inside the nonlocal medium. It is absent in the LAM computation as the longitudinal penetration depth is considered infinitely small (local model of permittivities) and only the transverse components of the EM-fields are considered. However, the introduction in LAM of a thin analogue layer at $y = 0\ \mu m$ of permittivity $\epsilon_{LAM}(\omega)$ given above allows to reproduce very well both the magnitude and decay of the EM-fields away from the interface obtained in the rigorous computation. This validates the equivalence between the two models.

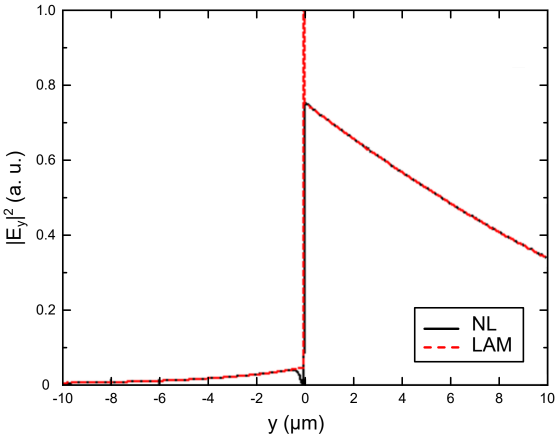


**Supplementary Figure 3:** Profile $|E_y|^2$ of the electric field normal to the interface for a TM-polarized plane wave incident at 80° on a planar $Si_3N_4$/InSb interface. The interface is located at $y = 0\ \mu m$ on the graph. The computation is performed for a rigorous nonlocal computation ("NL", black solid line) and for a simulation within the LAM ("LAM", red dashed curve). Parameters used in the simulations : $\omega_p = 2\pi\ 1.88\,(THz)$, $\omega = 2\pi\ 1.2\,(THz)$, $\Gamma^* = 2\pi\ 0.26\,(THz)$, $\epsilon_\infty = 17.5$, $\epsilon_{Si_3N_4} = 6.1$, $\epsilon_{LAM} = (1.2 - 2.6802i) * 10^{-5}$, $h = 10^{-6}\ \mu m$, $v_F = 10^6\ m.s^{-1}$.

We present in Supplementary Fig. 4 the best fits obtained from the nonlocal EM-simulations based on LAM used to derive the nonlocal parameter $v_F$ for the smallest stripe **m/i/p** cavity sample presented in the main text (Sample 4, $s = 5.5\ \mu m$). We display the experimental uncertainty on the absolute value of the absorption together with the simulated absorption spectra of the local EM-model for comparison.

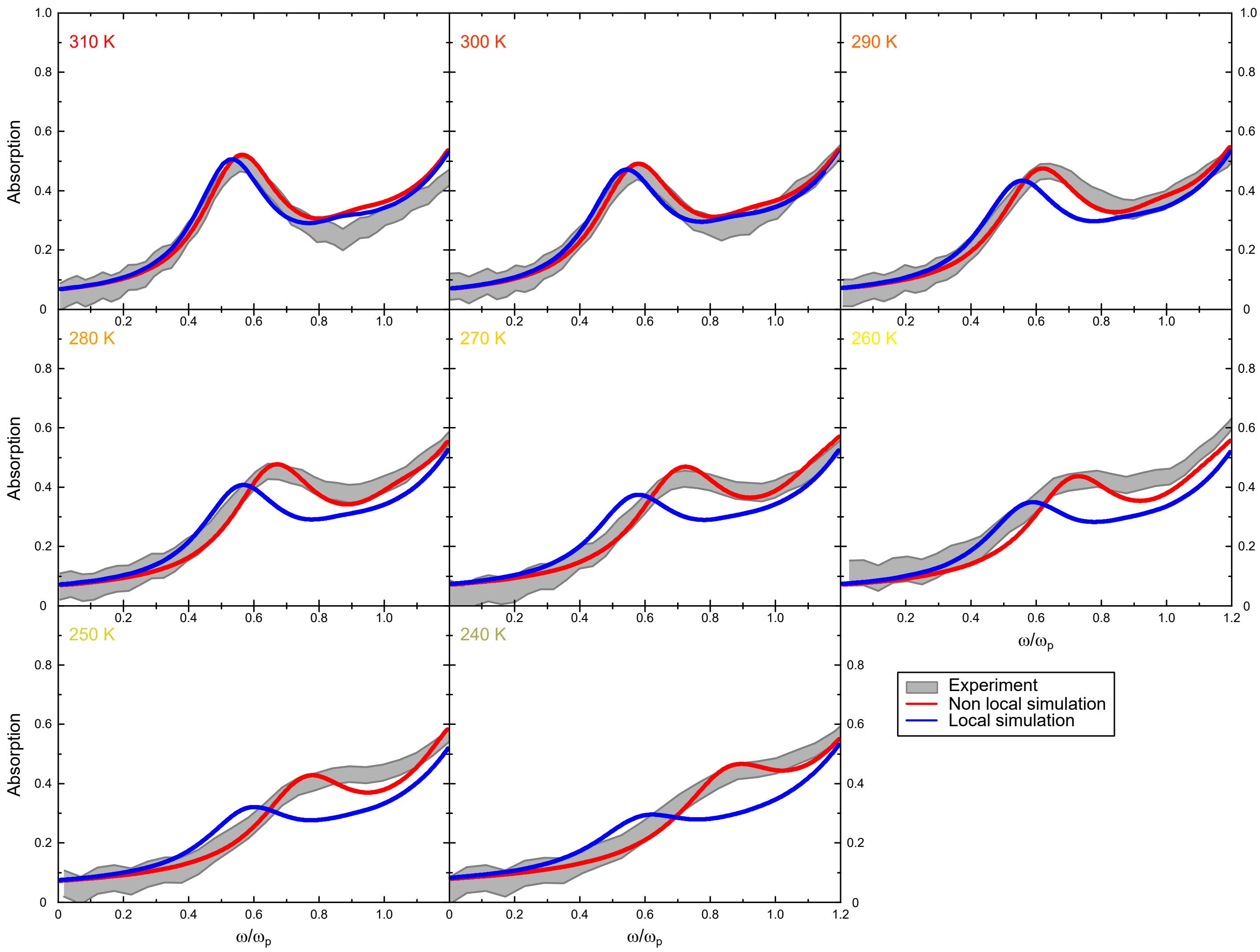


**Supplementary Figure 4:** Absorption spectra as a function of temperature for the smallest stripe **m/i/p** cavity (sample 4). Absolute values of the experimental absorption and its uncertainty (grey area) are displayed together with parameter free simulations within a local EM-model (blue solid line) and the best fits using a nonlocal model for the $v_F$ parameter estimation (red solid line). Experimental uncertainty (grey area) represents a statistical 10% confidence interval on the absorption value estimated from different measurements runs.

# 2 Patch m/i/p THz cavity

Below we present the experimental results obtained for patch **m/i/p** resonators and compare it to the other cavity architectures investigated in this study.

## 2.1 Patch m/i/p cavity experimental results

As opposed to stripe **m/i/p** cavities where the metallic stripes are infinite along the $z$-direction, patch cavities have a finite extent along both $z$ and $x$ directions. This allows to determine the three dimensional THz mode volume reported in the main text. We present in Supplementary Fig. 5 the experimental results for a patch **m/i/p** cavity sample manufactured with the same procedure as used for the striped **m/i/p** cavities. Due to the four-fold symmetry of the structure, cavity modes are optically active and degenerate for both $x$- and $z$-polarized THz electric field. The absolute reflectivity was obtained in this case by referencing it to a plain Gold coated substrate. Overall, the **m/i/p** patch cavity exhibit resonance characteristics that are very much similar, both at room temperature and in their temperature dependence, to the resonance characterisctics of the smallest stripe **m/i/p** cavity (Sample 4, $s = 5.5\ \mu m$) that has commensurate geometrical parameters.

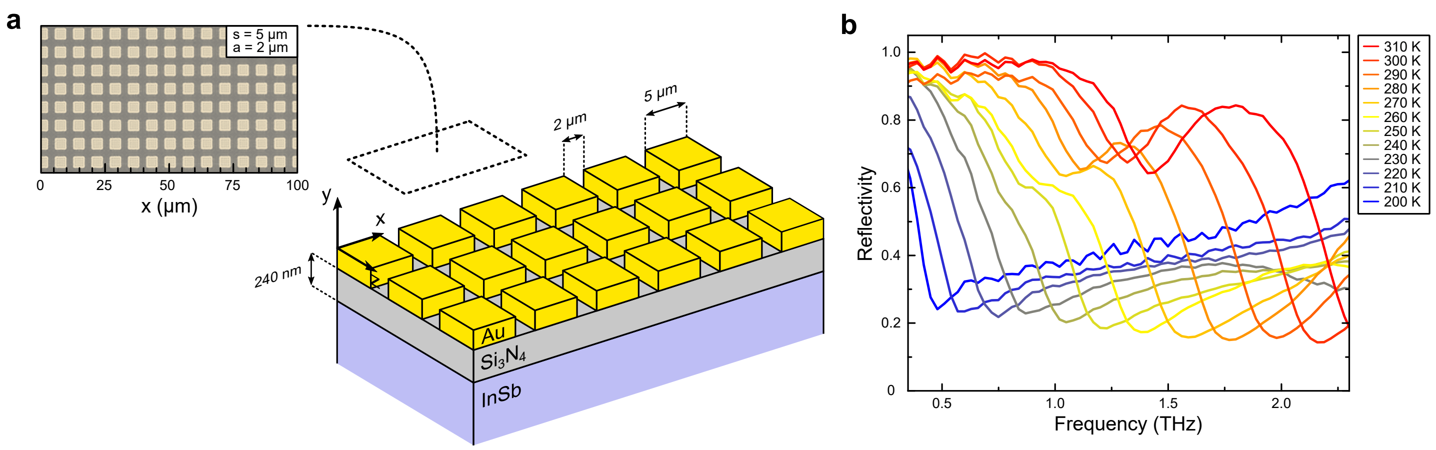


**Supplementary Figure 5:** **a**, Patch **m/i/p** cavity sample architecture and top view optical picture of the sample. **b**, Temperature dependence of the reflectivity spectra of the sample.

## 2.2 THz light confinement from stripe m/i/m to patch m/i/p resonators

Below we compare together the confinement properties of the different resonators investigated in this study at a common temperature (T = 300 K). Despite having commensurate resonance frequencies located in the 1-1.3 THz range, the different cavities span a wide range of geometrical parameters. As expected, the stripe **m/i/m** cavities are the largest ones. As compared to the latter, the role of the THz plasma and its ability to upper-bound the cavity frequencies by $\nu_p$ is strikingly evidenced by the possibility to shrink considerably the sizes of the resonators in all three dimensions while keeping their resonance frequencies almost unaffected. Such a plasmonically induced down-scaling effect is reported in Supplementary Fig. 6.

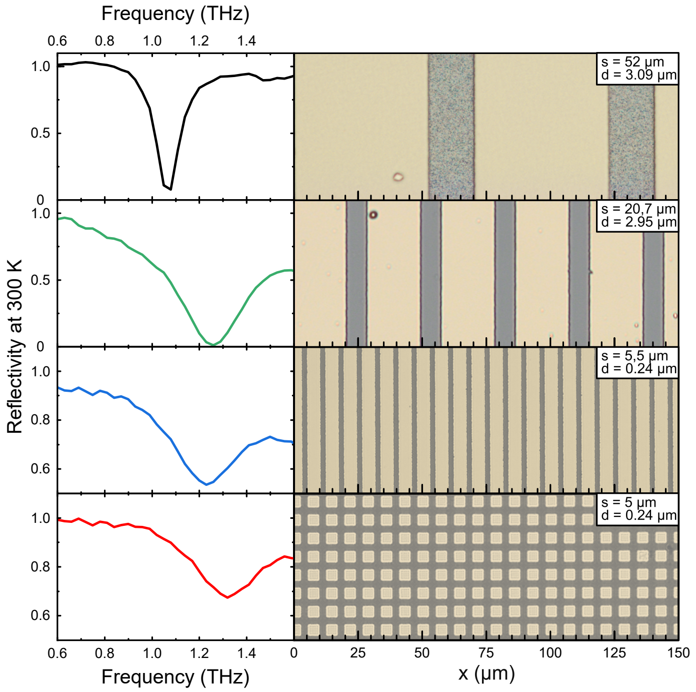


**Supplementary Figure 6:** Reflectivity spectra at T=300K for different cavity architectures with increasing confinement properties (left panels) and top view optical pictures of the samples (right panels). From top to bottom: **m/i/m** stripe cavity (sample 6), large **m/i/p** stripe (sample 3), small **m/i/p** stripe (sample 4), **m/i/p** patch cavity (sample 5). The spatial scale is identical in all pictures for comparison.

In the table below, we report the total, radiative and non-radiative quality factors (respectively $Q_{tot}$,$Q_{rad}$ and $Q_{ohm}$) of these cavity arrays based on the procedure described in [9] ($Q-$factors are defined here as $Q = \nu_0/\Delta\nu$). We note that the total quality factors are on the order of 3 to 4 for all plasmonic cavities and that they are limited by the non-radiative quality factor due to plasmonic losses. The metallic **m/i/m** cavity array achieves a slightly better quality factor of $\sim$ 9 but lies

| Type | Stripe **m/i/m** | Stripe **m/i/p** | Stripe **m/i/p** | Patch **m/i/p** |
|---|---|---|---|---|
| $s/d$ | 52.0 $\mu m$/3.09 $\mu m$ | 20.7 $\mu m$/2.95 $\mu m$ | 5.5 $\mu m$/0.24 $\mu m$ | 5 $\mu m$/0.24 $\mu m$ |
| $Q_{rad}$ | 24.6 | 5.9 | 23.1 | 37.3 |
| $Q_{ohm}$ | 14 | 5.9 | 3.6 | 3.7 |
| $Q_{tot}$ | 8.9 | 3.0 | 3.1 | 3.4 |

**Supplementary Table 2:** $Q-$factors of the samples

in the same range as for the plasmonic cavity arrays. While we chose here to match as much as possible the radiative and non-radiative quality factors in order to obtain a large contrast for the cavity resonances, quality factors can tuned away from critical coupling with the geometrical parameters of the array, which may be beneficial depending on the desired application [10].

# 3 Analysis of THz plasmonic modes in periodic lattices of m/i/p cavities

In this section, we analyze the major characteristics of the resonances found in our structures. We also give an intuitive picture regarding the origin and nature of these resonances.

## 3.1 Dispersion diagram

We present below the dispersion diagram of the structure computed for the sample #1. This dispersion diagram reports the frequencies of the major resonances of the structure as a function of incident angle $\theta$ of the light impinging onto the structure. The actual structure being periodic of period $s + a$, the dispersion is plotted in the first Brillouin zone. A few remarks can

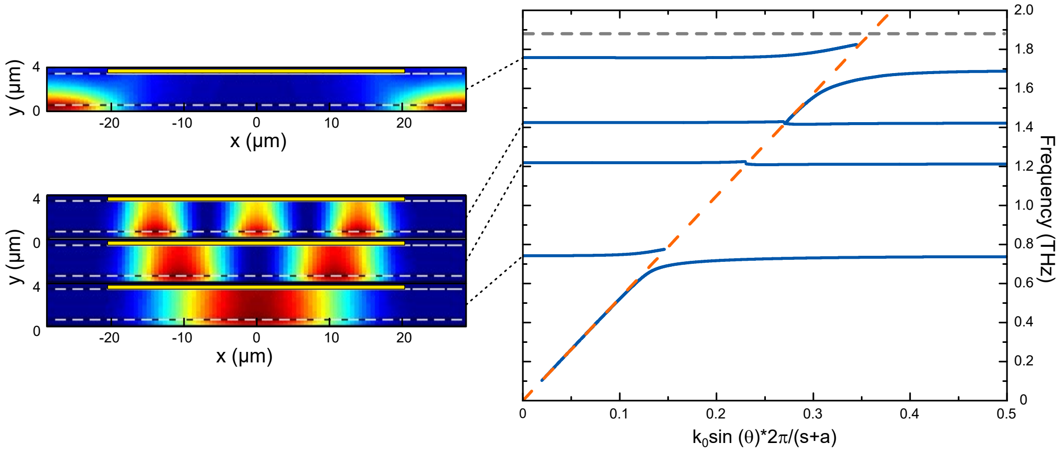


**Supplementary Figure 7:** Dispersion diagram of the sample #1: frequencies of the major resonances as a function of incident angle $\theta$ of the light impinging onto the structure. The resonances are depicted as blue solid lines, the orange dashed line corresponds to the dispersion of the light in vacuum, the grey dashed line is the plasma frequency $\nu_p$, $k_0$ is the wavevector of the light in vacuum. The modes associated with these resonances are shown for $\theta = 0°$. Parameters used for the calculation: $\omega_p = 2\pi\, 1.88\,(THz)$, $\Gamma^* = 2\pi\, 0.26\,(THz)$, $\epsilon_\infty = 17.5$, $\epsilon_i = 6.1$, which correspond to our material parameters at T=290K. The geometrical parameters are those of sample 1.

be made regarding its interpretation:

- Our experiment is performed at normal incidence and the resonance frequencies measured experimentally can be obtained on this diagram at $\theta = 0°$.
- By looking at the mode profiles of these resonances (see Supplementary Fig. 7), we find that the first three resonances at $\nu \approx 0.7$ THz, 1.2 THz and 1.4 THz are associated with modes located in the **m/i/p** region below the metallic stripe. They correspond to the fundamental, second and third harmonic of the **m/i/p** cavity resonances respectively. By symmetry, the second resonance cannot be excited at normal incidence and we do not observe it experimentally. We observe that each of these resonances is almost dispersionless as a function of the angle $\theta$. This signals the fact that they correspond to strongly localized modes of the structure.

- The resonance at $\nu \approx 1.8$ THz for $\theta = 0°$ corresponds to a mode located in the **vacuum/i/p** region. It is the fundamental resonance of the inter-cavity region. We observe that this resonance emerges from a resonance that is initially strongly dispersive as it follows the dispersion relation of the light in vacuum before the resonance eventually saturates and gets folded back by the periodicity of the structure. After the folding, the resonance is almost dispersionless as a function of $\theta$, which signals a strongly localized mode of the structure.

## 3.2 Interpretation based on Fabry-Perot resonances of m/i/p and vacuum/i/p waveguides

Here, we present an interpretation of the resonance frequencies of the actual structure as ensuing from the dispersion relations of two separate entities: (i) a **m/i/p** waveguide and (ii) a **vacuum/i/p** waveguide. To that end, we compare below the dispersion relations of these two waveguides together with the resonance frequencies of the actual structure obtained at normal incidence ($\theta = 0°$) and by considering additional branches in the dispersion diagram of Supplementary Fig. 7. Both are plotted as a function of $k_x$. For the dispersion relations, $k_x$ is simply the wavevector along the direction of propagation inside the waveguides. For the resonances of the actual structure, this quantity is assumed to take the following functional form: (i) $k_x = n\pi/s$ for the resonances located in the **m/i/p** region and (ii) $k_x = n\pi/a$ for the resonances located in the **vacuum/i/p** region ($n = 1, 2...$ being the number of maxima of the magnetic field of the mode). We observe that the functional form

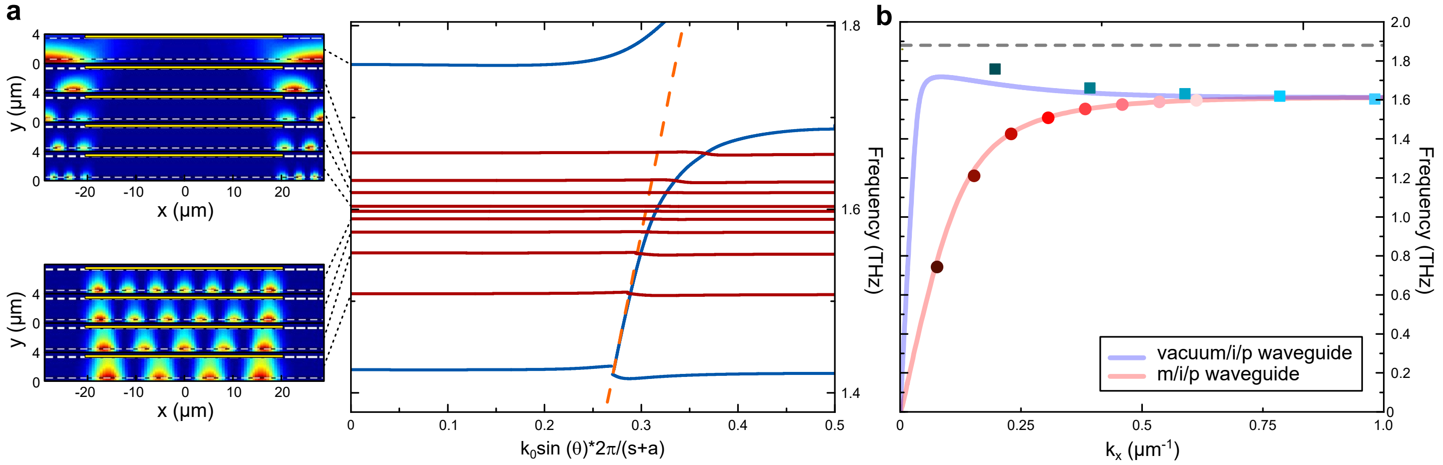


**Supplementary Figure 8:** **a**, Dispersion diagram of the structure presented with additionnal branches (red solid lines) as compared to the major branches already shown in Supplementary Fig. 7 (blue solid lines). The modes associated with these resonances are shown for $\theta = 0°$. **b**, Dispersion relations of the **m/i/p** (red solid line) and **vacuum/i/p** (blue solid line) waveguides. They are compared with the resonance frequencies (colored data points) of the actual structure obtained from the dispersion diagram at $\theta = 0°$. Resonance frequencies are plotted as a function of $k_x$ assumed to have the following form: (i) $k_x = n\pi/s$ for the resonances located in the **m/i/p** region and (ii) $k_x = n\pi/a$ for the resonances located in the **vacuum/i/p region** ($n = 1, 2...$ being the number of maxima of the magnetic field of the mode). Material parameters used for the computation in both cases: $\omega_p = 2\pi\, 1.88\, (THz)$, $\Gamma^* = 2\pi\, 0.26\, (THz)$, $\epsilon_\infty = 17.5$, $\epsilon_i = 6.1$. Geometrical parameters for the simulation of the actual structure are those of Sample 1. The thickness of the insulator taken to compute the dispersion relations of the waveguides is that of Sample 1 ($d = 2.95\mu m$).

chosen for the wavector $k_x$ allows the resonances of the actual structure to be very well described by the dispersion relations of the corresponding waveguides. The picture that emerges from this agreement is that both the cavity and inter-cavity regions act as Fabry-Perot resonators for THz surface plasmons modes although these modes have opposite dispersions. This arises mostly due to the localized nature of the cavity and inter-cavity resonances in the structure. While this could be anticipated for the cavity modes, it is less obvious in the case of the inter-cavity modes. However, we note that at the wavevectors considered here, resonances frequencies of the inter-cavity modes fall into the photonic bandgap of the **m/i/p** waveguide. Hence, the neighbouring **m/i/p** cavities act as efficient "mirrors" allowing the inter-cavity mode to be confined and to resonate. Hence, the geometrical parameters $s$ and $a$ select the wavector $k_x$ of the mode and the corresponding resonance frequencies are to a good extent identical to that of the propagating waveguide mode with same wavector. This allows to explain remarkably well the scaling of the resonance frequencies as a function of the geometrical parameters $s$ and $a$ shown in Fig. 2c of the main text.

Regarding the inter-cavity resonance however, we observe from the full-wave simulations that the fundamental inter-cavity mode has a finite penetration depth into the neighbouring **m/i/p** cavities. The effective size of the mode and its wavector are not simply defined by the geometrical parameter $a$ alone in this case. We write the wavevector as $k_x = \frac{\pi}{a+2\delta}$ where $\delta$ is the penetration depth of the mode inside the adjacent **m/i/p** cavities. By fitting the magnetic field profiles of the modes obtained from the full-wave simulations, we find that $9\mu m < \delta < 10\mu m$ for the fundamental inter-cavity mode of all three

largest cavities (samples 1, 2 and 3). The corresponding $k_x$ are the ones used for the plot in Fig. 2c of the main text.

### 3.3 Intuitive picture

In this part, we describe an intuitive picture allowing to understand simply the major characteristics of the plasmonic resonances found in our structures, such as frequencies, dispersions and mode profiles. We show that despite the dissymetry and periodicity of the actual structure, these characteristics are very much similar to the characteristics of the plasmonic modes of an analogue symmetric and infinite **p/i/p** waveguide. We relate this equivalence to the symmetries enforced onto the modes' structures that ensue from the boundary conditions imposed at the top insulator interface.

To show this, we start by recalling the dispersion relation of symmetric and infinite **p/i/p** waveguide, which can be found for instance in [8]:

$$e^{2k_i d^*} = r_p^2 \tag{1}$$

Here, we consider a plasmonic material with permittivity $\epsilon_p(\omega) = \epsilon_\infty \left(1 - \frac{\omega_p^2}{\omega(\omega+i\Gamma^*)}\right)$, an insulator of permittivity $\epsilon_i$ and thickness $d^*$, $r_p = \frac{k_i\epsilon_p(\omega)-k_p\epsilon_i}{k_i\epsilon_p(\omega)+k_p\epsilon_i}$ is the reflection coefficient at the **p/i** interface, $k_p = \sqrt{k_x^2 - \frac{\omega^2}{c^2}\epsilon_p(\omega)}$ and $k_i = \sqrt{k_x^2 - \frac{\omega^2}{c^2}\epsilon_i}$. This equation has two solutions:

- The first solution is:

$$e^{k_i d^*} = +r_p \tag{2}$$

  This solution describes the symmetric plasmon mode ("bonding mode") of symmetric waveguides. The symmetric mode is characterized by a symmetric mode profile of the magnetic field with respect to the plane of symmetry of the waveguide located at $d^*/2$

- The second solution is:

$$e^{k_i d^*} = -r_p \tag{3}$$

  This solution describes the antisymmetric plasmon mode ("antibonding mode") of symmetric waveguides. In this case, the magnetic field vanishes at the plane of symmetry of the waveguide located at $d^*/2$.

The resulting dispersion relations and the corresponding mode profiles are shown in Supplementary Fig. 9. For a dissymetric

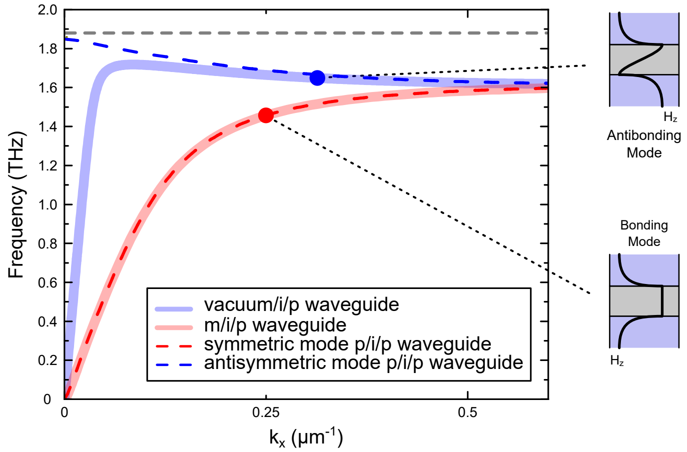


**Supplementary Figure 9:** Dispersion relations of the symmetric (red dashed line) and antisymmetric (blue dashed line) modes of a symmetric **p/i/p** waveguide with insulator thickness $d^* = 2 * 2.95\ \mu m = 5.90\ \mu m$. Sketches of the magnetic field profile are shown for the two types of modes. The dispersion relations are compared with that of the **m/i/p** (red solid line) and **vacuum/i/p** (blue solid line) waveguides shown in Supplementary Fig. 8b with insulator thickness $d = 2.95\mu m$. In both cases, material parameters are: $\omega_p = 2\pi\, 1.88\, (THz)$, $\Gamma^* = 2\pi\, 0.26\, (THz)$, $\epsilon_\infty = 17.5$, $\epsilon_i = 6.1$, which correspond to our material parameters at temperature T=290K.

waveguide **p'/i/p** composed of two different plasmas $p$ and $p'$ separated by an insulator of thickness $d$, it is easy to show that the dispersion relation (1) must be generalized to :

$$e^{2k_i d} = r_{p'} r_p \tag{4}$$

where $r_\alpha = \frac{k_i \epsilon_\alpha(\omega) - k_\alpha \epsilon_i}{k_i \epsilon_\alpha(\omega) + k_\alpha \epsilon_i}$ and the subscript $\alpha = p, p'$ refers to the reflection coefficient at the **p/i** and **p'/i** interfaces respectively. The permittivities are $\epsilon_\alpha(\omega) = \epsilon_{\infty,\alpha}\left(1 - \frac{\omega_\alpha^2}{\omega(\omega + i\Gamma_\alpha^*)}\right)$ and $k_\alpha = \sqrt{k_x^2 - \frac{\omega^2}{c^2}\epsilon_\alpha(\omega)}$.

For the cavity mode located in the **m/i/p** region below the metallic stripe, the metal can be described by the plasma $p'$ with a large plasma frequency ($\omega_{p'} >> \omega_p$, $\omega_{p'}$ being the plasma frequency of the metal). For frequencies in the THz range, we have that $r_{p'} \approx 1$ and the metal acts similarly to a perfect electric conductor (PEC) for which the magnetic field of the wave does not experience any phase shift upon reflection at the **m/i** boundary (on the contrary to the electric field which experiences a $\pi$ phase shift). The resulting dispersion relation (4) becomes: $e^{2k_i d} \approx +r_p$. It is identical to the dispersion relation (2) of the symmetric mode of an analogue symmetric **p/i/p** waveguide, albeit that the thickness of the insulator $d^*$ of the analogue **p/i/p** waveguide should be taken as twice the thickness $d$ of the **m/i/p** waveguide: $d^* = 2d$. The perfect equivalence between the two dispersions is evident in Supplementary Fig. 9. Additionally, from the PEC boundary condition imposed at the **m/i** interface, the magnetic field is maximized at this interface located at distance $d$ from the plasma $p$. The same condition holds for the symmetric mode of the analogue **p/i/p** waveguide as it exhibits a maximum of the magnetic field at the symmetry plane located at $d^*/2 = d$. In other words, the metallic stripe acts as a PEC mirroring the plasma $p$ and the cavity mode profile of the actual structure is identical to that of the symmetric mode of the analogue waveguide. In turn, we can consider that enforcing such symmetry onto the mode's structure is the true origin of the correspondence between the two dispersion relations.

For the inter-cavity mode located in the **vacuum/i/p** region, the reflection takes place from the dielectric to the vacuum. The vacuum can be described by the plasma $p'$ by taking $\epsilon_{\infty,p'} = 1, \omega_{p'} = \Gamma_{p'}^* = 0$. If the dielectric contrast is sufficiently large between the insulator and the vacuum ($\epsilon_i >> 1$), this results in $r_{p'} \approx -1$ as the magnetic field acquires a phase shift close to $\pi$ upon reflection. This selects the dispersion relation (3) ($e^{2k_i d} \approx -r_p$) identical to that of the antisymmetric mode (antibonding mode) of an analogue **p/i/p** symmetric waveguide with the thickness of the insulator of the waveguide being $d^* = 2d$ as before. In this case, the **vacuum/i** interface acts like a Perfect Magnetic conductor (PMC) and imposes a cancellation of the magnetic field at that location. The very same condition holds true for the antisymmetric mode of the analogue **p/i/p** waveguide, where, by symmetry, the magnetic field is null at the symmetry plane located at $d^*/2 = d$. Hence, the mode profile of the inter-cavity mode ensues from the mode profile of the antisymmetric mode of the analogue waveguide. We note that in the present experimental case, the PMC condition is not fully reached as $r_{p'} = \frac{1-\sqrt{\epsilon_i}}{1+\sqrt{\epsilon_i}} \approx -0.42$ so that using a dielectric with larger permittivity would help reaching the PMC boundary condition. In Fig. 2d of the main text, it is indeed observed that the magnetic field of the inter-cavity mode gets strongly reduced close to the **vacuum/i** interface but that it does not cancel out completely. The good correspondence between the **vacuum/i/p** and analogue **p/i/p** waveguides is shown in Supplementary Fig. 9 for our parameters. The difference between the two dispersions observed at small wavevectors is easily explained by the incomplete PMC condition so that the assumption of the cancellation of the magnetic field at the **vacuum/i** interface used to obtain the equivalence is breaking down.

Overall, such a simple picture based on the symmetries imposed onto the modes' structures at the top insulator interace allows to explain well the resonances frequencies, dispersions and mode profiles of the cavity and inter-cavity resonances found experimentally.

This analysis is summarized in Supplementary Fig. 10 where the magnetic field profiles of both cavity and inter-cavity

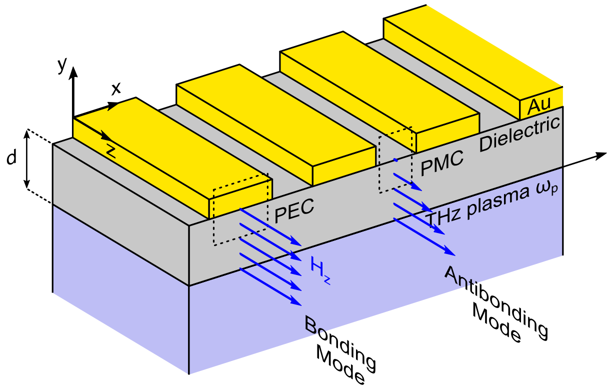


**Supplementary Figure 10:** Sketch of the magnetic field profiles of the cavity and inter-cavity modes within the insulator region only.

modes are sketched within the insulating region only. Finally, let's note that an interesting result that comes out from this analysis and confirmed experimentally is the possibility to excite THz surface plasmons with negative dispersion at a *single*

dielectric/plasma interface. These plasmons with negative dispersion can be designed from a suitable choice of insulator's permittivity and thickness and they can be excited with a metallic coupler as done here.

# Supplementary References


[1] Chochol, J. et al. Plasmonic behavior of III-V semiconductors in far-infrared and terahertz range. Journal of the European Optical Society-Rapid Publications **13** (2017).

[2] Todorov, Y. et al. Optical properties of metal-dielectric-metal microcavities in the THz frequency range. Optics Express **18**, 13886 (2010).

[3] Laman, N. & Grischkowsky, D. Terahertz conductivity of thin metal films. Applied Physics Letters **93**, 051105 (2008).

[4] Tomasino, A. et al. Invited article: Ultra-broadband terahertz coherent detection via a silicon nitride-based deep sub-wavelength metallic slit. APL Photonics **3**, 110805 (2018).

[5] Cataldo, G. et al. Infrared dielectric properties of low-stress silicon nitride. Optics Letters **37**, 4200 (2012).

[6] Luo, Y., Fernandez-Dominguez, A. I., Wiener, A., Maier, S. A. & Pendry, J. B. Surface plasmons and nonlocality: A simple model. Physical Review Letters **111**, 093901 (2013).

[7] Sakat, É., Moreau, A. & Hugonin, J.-P. Generalized electromagnetic theorems for nonlocal plasmonics. Physical Review B **103**, 235422 (2021).

[8] Moreau, A., Ciracì, C. & Smith, D. R. Impact of nonlocal response on metallodielectric multilayers and optical patch antennas. Physical Review B **87**, 045401 (2013).

[9] Manceau, J.-M., Zanotto, S., Sagnes, I., Beaudoin, G. & Colombelli, R. Optical critical coupling into highly confining metal-insulator-metal resonators. Applied Physics Letters **103** (2013).

[10] Feuillet-Palma, C., Todorov, Y., Vasanelli, A. & Sirtori, C. Strong near field enhancement in THz nano-antenna arrays. Scientific Reports **3** (2013).
